# Factors Associated with Reoperation in Breast-Conserving Surgery for Cancer: A Prospective Study of American Society of Breast Surgeon Members

**DOI:** 10.1245/s10434-019-07547-w

**Published:** 2019-07-24

**Authors:** Jeffrey Landercasper, Andrew J. Borgert, Oluwadamilola M. Fayanju, Hiram Cody, Sheldon Feldman, Caprice Greenberg, Jared Linebarger, Barbara Pockaj, Lee Wilke

**Affiliations:** 1 Norma J. Vinger Center for Breast Cancer, Gundersen Health System, La Crosse, WI USA; 2 Department of Medical Research, Gundersen Medical Foundation, La Crosse, WI 54601 USA; 3 Department of Surgery, Duke University Medical Center, Durham, NC USA; 4 Department of Surgery, Memorial Sloan Kettering Cancer Center, New York, NY USA; 5 Montefiore Einstein Center for Cancer Care, Montefiore Medical Center, Bronx, NY USA; 6 University of Wisconsin School of Public Health and Medicine, Madison, WI USA; 7 Department of Surgery, Gundersen Health System, La Crosse, WI USA; 8 Department of Surgery, Mayo Clinic, Phoenix, AZ USA

## Abstract

**Background:**

More than 20% of patients undergoing initial breast-conserving surgery (BCS) for cancer require reoperation. To address this concern, the American Society of Breast Surgeons (ASBrS) endorsed 10 processes of care (tools) in 2015 to be considered by surgeons to de-escalate reoperations. In a planned follow-up, we sought to determine which tools were associated with fewer reoperations.

**Methods:**

A cohort of ASBrS member surgeons prospectively entered data into the ASBrS Mastery^®^ registry on consecutive patients undergoing BCS in 2017. The association between tools and reoperations was estimated via multivariate and hierarchical ranking analyses.

**Results:**

Seventy-one surgeons reported reoperations in 486 (12.3%) of 3954 cases (mean 12.7% [standard deviation (SD) 7.7%], median 11.5% [range 0–32%]). There was an eightfold difference between surgeons in the 10th and 90th percentile performance groups. Actionable factors associated with fewer reoperations included routine planned cavity side-wall shaves, surgeon use of ultrasound (US), neoadjuvant chemotherapy, intra-operative pathologic margin assessment, and use of a pre-operative diagnostic imaging modality beyond conventional 2D mammography. For patients with invasive cancer, ≥ 24% of those who underwent reexcision did so for reported margins of < 1 or 2 mm, representing noncompliance with the SSO-ASTRO margin guideline.

**Conclusions:**

Although ASBrS member surgeons had some of the lowest rates of reoperation reported in any registry, significant intersurgeon variability persisted. Further efforts to lower rates are therefore warranted. Opportunities to do so were identified by adopting those processes of care, including improved compliance with the SSO-ASTRO margin guideline, which were associated with fewer reoperations.

Reoperations after initial breast-conserving surgery (BCS) for cancer are common, and rates vary significantly between surgeons and facilities.^1^^–^17 Rates of reoperation average approximately 20% and range from less than 10% to more than 60%. As a result, multiple stakeholders have undertaken initiatives to reduce rates.^18^^–^27 For example, after a meta-analysis, the Society of Surgical Oncology (SSO) and the American Society for Radiation Oncology (ASTRO) convened a consensus conference in 2013, which proposed a guideline that recommended that surgeons omit reexcisions to achieve margins wider than “no ink on tumor” for patients undergoing BCS for invasive cancer.^19^,^20^ The American Society of Breast Surgeons (ASBrS) and others subsequently endorsed this margin guideline.^18^,^27^,^28^ In a parallel effort to reduce rates, the ASBrS convened a multidisciplinary conference in 2015. During this “Collaborative Attempt to Lower Lumpectomy Re-Excisions” (CALLER), conference participants reviewed more than 100 publications relevant to reoperations and then recommended 10 processes of care to serve as tools surgeons could potentially employ to reduce rates of reexcision.^18^

Subsequent to the CALLER Conference, a prospective study of reoperation rates reported by ASBrS member-surgeons was conducted. The primary purpose was to determine the efficacy of the tools in the CALLER toolbox at lowering rates of reoperation in patients undergoing BCS for cancer.

## Methods

The principal investigator’s (PI) Institutional Review Board (IRB), the ASBrS Research Committee, selected members of the ASBrS Patient Safety and Quality Committee, and study co-investigators approved the study design.

Surgeons signed an attestation document to prospectively enter information into the ASBrS patient registry (Mastery^®^) on consecutive patients undergoing initial BCS. Surgeons received a $500.00 stipend. Participation was voluntary. Recruitment occurred by an ASBrS newsletter and member email.

### Patients

**Fig. 1:**
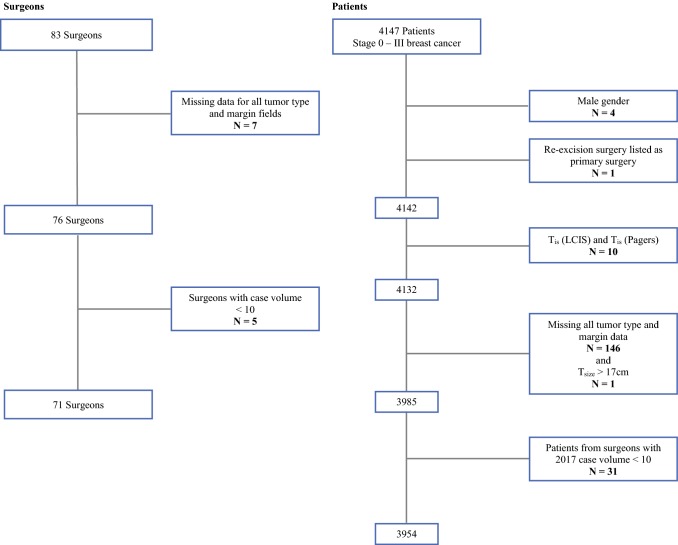
Patients and surgeons

Patients included those undergoing BCS initially or after neoadjuvant treatment for Stage 0–III breast cancer in 2017 (Fig. 1). All patients had a preoperative diagnosis of malignancy by a core needle biopsy or other minimally invasive technique.

One surgeon provided transparency of one patient to the PI by nonpassword-protected email. After institutional review board notification, the patient was excluded.

### Independent variables

Univariate tests of association and model components included in the final multivariate (MV) model of risk of reoperation are shown in Table 1. Multiple different uses of breast ultrasound were treated as independent variables.

**Table 1:** Association of patient, tumor, process of care, and surgeon practice characteristics with reoperations after breast conserving surgery for breast cancer, CALLER Registry, American Society of Breast Surgeons Mastery^®^ database

	Univariate				Multivariate^a^			
	Receipt of reoperation (*n *= 486)	Total (*n *= 3954)	Rate (12.3%)	*p* value	Odds ratio	95% CI		*p* value
						Lower	Upper	
*Patient*								
Age (yr)								
80+	24	344	7	< 0.001	Reference			
70–79	116	988	11.7		2.22	1.27	3.86	0.005
60–69	178	1288	13.8		1.94	1.19	3.17	0.008
50–59	91	859	10.6		1.90	1.17	3.09	0.010
< 50	77	475	16.2		1.25	0.74	2.13	0.408
Race								
African American	50	323	15.5	0.200				
Caucasian/Hispanic	425	3530	12					
Other	31	250	12.4					
Primary insurance								
Medicare traditional	125	1319	9.5	0.002	Reference			
Commercial	319	2247	14.2		1.58	1.19	2.09	0.002
Medicaid/state-managed	23	187	12.3		1.22	0.69	2.16	0.488
Tricare	6	42	14.3		0.79	0.24	2.58	0.691
No insurance	4	24	16.7		2.36	0.67	8.25	0.180
Unknown	29	283	10.3		1.02	0.56	1.84	0.956
Missing	0	1	0					
*Tumor*								
Surgery side								
Left	253	2114	12	0.470				
Right	253	1989	12.7					
Mean size ± standard deviation (mm)^b^	No reexcision: 15.4 ± 11.4; Reexcision: 18.8 ± 13.8			< 0.001	1.02	1.01	1.03	< 0.001
pT stage								
T1mi/a/b	88	1175	7.5	< 0.001				
T1c	143	1279	11.2					
T2	102	611	16.7					
T3/T4	8	36	22.2					
Tis (DCIS)	135	636	21.2					
TX	1	88	1.1					
Missing	29	278	10.4					
pN stage								
N0	329	2892	11.4	0.003				
N1	51	364	14					
N2	12	59	20.3					
N3	2	16	12.5					
NX	83	494	16.8					
Missing	29	278	10.4					
Histology								
IDC	127	1651	7.7	< 0.001				
IDC and ILC	14	89	15.7					
ILC	44	266	16.5					
IDC and DCIS	156	1105	14.1					
Other	5	91	5.5					
DCIS only	131	633	20.7					
Missing	29	268	10.8					
Focality								
Unifocal	348	3358	10.4	< 0.001	Reference			
Multifocal/multicentric	124	444	27.9		3.64	2.77	4.78	< 0.001
Uncertain	5	31	16.1		2.85	0.88	9.19	0.080
Stage and histology composite^c^								
T1mi/a/b IDC	35	613	5.7	< 0.001	Reference			
T1mi/a/b ILC	3	77	3.9		0.62	0.18	2.14	0.448
T1mi/a/b Mixed/other	50	484	10.3		1.86	1.15	3.01	0.011
T1c IDC	55	659	8.4		1.42	0.90	2.26	0.134
T1c ILC	13	109	11.9		1.67	0.81	3.44	0.162
T1c Mixed/other	75	510	14.7		2.63	1.67	4.14	< 0.001
T2 IDC	31	295	10.5		1.53	0.89	2.65	0.127
T2 ILC	25	69	36.2		7.84	4.04	15.21	< 0.001
T2 Mixed/other	46	247	18.6		3.12	1.86	5.24	< 0.001
T3/T4 All types	8	36	22.2		2.15	0.79	5.83	0.134
TX	1	88	1.1		0.21	0.03	1.65	0.137
Tis (DCIS)	135	636	21.2		3.71	2.43	5.67	< 0.001
Missing	29	280	10.4					
*Surgeon practice setting/characteristic*								
Practice type								
Solo private practice	90	1013	8.9	< 0.001	Reference			
Academic	30	251	12		0.92	0.39	2.17	0.843
Group private practice	268	1650	16.2		2.31	1.45	3.69	< 0.001
Hospital employed practice	118	1189	9.9		1.60	0.95	2.70	0.075
Length of time in practice								
10 years or less	145	1101	13.2	0.009				
11–20 years	167	1371	12.2					
21–30 years	170	1280	13.3					
More than 30 years	24	351	6.8					
Proportion of practice breast surgery								
100%	367	3197	11.5	0.005				
> 50%	122	763	16					
25–50%	13	90	14.4					
< 25%	4	53	7.6					
I perform breast ultrasound^d^								
No	105	681	15.4	0.007	Reference			
Yes	401	3422	11.7		0.57	0.35	0.92	0.022
I perform ultrasound-guided office breast procedures^d^								
No	136	901	15.1	0.004				
Yes	370	3202	11.6					
I perform ultrasound-guided breast procedures in the operating room^d^								
No	136	1025	13.3	0.300				
Yes	370	3078	12					
I perform stereotactic-guided breast procedures								
No	318	2461	12.9	0.150				
Yes	188	1642	11.5					
Surgeon per annum case volume in top quartile								
No	301	2173	13.9	0.001	Reference			
Yes	205	1930	10.6		0.68	0.48	0.98	0.038
National Consortium of Breast Centers participation								
No	412	3146	13.1	0.007				
Yes	94	957	9.8					
*Preoperative imaging modalities*								
2d diagnostic mammography								
No	313	2311	13.5	0.004	Reference			
Yes	173	1650	10.5		1.4	1.07	1.84	0.013
Missing	20	142	14.1					
Film mammography								
No	484	3947	12.3	0.810				
Yes	2	14	14.3					
Missing	20	142	14.1					
3d diagnostic mammography								
No	251	1907	13.2	0.100				
Yes	235	2054	11.4					
Missing	20	142	14.1					
Ultrasound (US)^d^								
No	114	651	17.5	< 0.001				
Yes	372	3310	11.2					
Missing	20	142	14.1					
Magnetic resonance imaging (MRI)								
No	295	2270	13	0.110				
Yes	191	1691	11.3					
Missing	20	142	14.1					
None								
No	486	3960	12.3	0.710				
Yes	0	1	0					
Missing	20	142	14.1					
*Radiologist*-*surgeon communication by report*								
Surgeon-radiology communication-did radiologist indicate size(s)?								
No	176	1434	12.3	0.990				
Yes	310	2527	12.3					
Missing	20	142	14.1					
Surgeon-radiology communication-did the radiologist indicate distance(s) to nipple, skin, other?								
No	426	3349	12.7	0.040				
Yes	60	612	9.8					
Missing	20	142	14.1					
*Breast conservation localization technique*								
Hematoma guided US^d^								
No	483	3881	12.5	0.019				
Yes	3	80	3.8					
Missing	20	142	14.1					
Ultrasound (US)^d^								
No	315	2327	13.5	0.004				
Yes	171	1634	10.5					
Missing	20	142	14.1					
Palpation								
No	410	3336	12.3	0.93				
Yes	76	625	12.2					
Missing	20	142	14.1					
Single wire								
No	253	2010	12.6	0.540				
Yes	233	1951	11.9					
Missing	20	142	14.1					
Multiple wires								
No	372	3330	11.2	< 0.001				
Yes	114	631	18.1					
Missing	20	142	14.1					
Any wire (simplified)								
No	146	1412	10.3	0.006				
Yes	340	2549	13.3					
Missing	20	142	14.1					
Radioactive seed(s)								
No	472	3840	12.3	0.810				
Yes	14	121	11.6					
Missing	20	142	14.1					
Magnetic resonance imaging (MRI)								
No	484	3946	12.3	0.900				
Yes	2	15	13.3					
Missing	20	142	14.1					
Mammography stereotactic								
No	386	3295	11.7	0.018				
Yes	100	666	15					
Missing	20	142	14.1					
SAVI SCOUT^®^ radar								
No	461	3757	12.3	0.990				
Yes	25	204	12.3					
Missing	20	142	14.1					
Other								
No	481	3926	12.3	0.610				
Yes	5	35	14.3					
Missing	20	142	14.1					
*Surgeon intraoperative practice (receipt)*								
Cavity side wall shaves performed?								
No	197	1383	14.2	< 0.001	Reference			
Selective based on intra-op findings	161	1225	13.1		0.86	0.64	1.15	0.299
Routine planned	127	1334	9.5		0.58	0.39	0.85	< 0.001
Missing	21	161	13					
Oncoplastic resection and any type closure								
No	359	2728	13.2	0.017	Reference			
Yes	124	1189	10.4		0.76	0.57	1.00	0.054
Missing	23	186	12.4					
Dune MarginProbe^®^ device								
No	486	3901	12.5	0.280				
Yes	20	202	9.9					
Margin evaluation type								
Beyond gross	19	122	15.6	0.001				
Gross	72	836	8.6					
None	395	3003	13.2					
Missing	20	142	14.1					
Any type of intra-operative pathologic margin assessment excluding margin device^e^								
Yes	91	965	9.4	0.002	0.66	0.46	0.95	0.027
No	395	2996	13.2		Reference			
Missing	20	142	14.1					
Gross evaluation margin								
No	410	3082	13.3	< 0.001				
Yes	76	879	8.7					
Missing	20	142	14.1					
Frozen selective margin(s)								
No	475	3902	12.2	0.130				
Yes	11	59	18.6					
Missing	20	142	14.1					
Touch-prep cytology margin								
No	477	3918	12.2	0.080				
Yes	9	43	20.9					
Missing	20	142	14.1					
Specimen orientation (# sides)^f^								
0	5	54	9.3	0.740				
1–2	120	997	12					
3 or more	361	2899	12.5					
Missing	20	153	13.1					
Ultrasound (US) by surgeon in operating room^d^								
No	448	3670	12.2	0.680				
Yes	38	291	13.1					
Missing	20	142	14.1					
Specimen mammography								
No	371	3208	11.6	0.005				
Yes	115	753	15.3					
Missing	20	142	14.1					
Specimen imaging single view								
No	218	1705	12.8	0.390				
Yes	268	2256	11.9					
Missing	20	142	14.1					
Specimen imaging multiple views								
No	316	2671	11.8	0.230				
Yes	170	1290	13.2					
Missing	20	142	14.1					
Was the specimen compressed for imaging?								
No	386	3245	11.9	0.070				
Yes	98	680	14.4					
Missing	22	178	12.4					
Guidance technique used?								
No image guidance	44	351	12.5	0.330				
Pre-op localization	135	1115	12.1					
Intra-op localization	32	342	9.4					
Image guidance	295	2295	12.9					
*Treatment*								
Receipt of neoadjuvant treatment								
No	448	3553	12.6	0.007	Reference			
Yes—Chemotherapy	27	356	7.6		0.45	0.28	0.73	0.001
Yes—Endocrine therapy	18	104	17.3		2.1	1.14	3.86	0.017
Missing	13	90	14.4					

*DCIS* ductal carcinoma in situ; *IDC* infiltrating ductal carcinoma; *ILC* invasive lobular carcinoma; *CI* confidence interval

^a^Covariates not significantly associated with reoperation, after accounting for the effects of other relevant covariates, were excluded from the final multivariate reoperation model by the stepwise selection process and are left blank in the right column

^b^Largest estimated pre-operative tumor size. The odds ratio for reexcision is for each 1 mm increase in tumor size

^c^Composite covariate to reflect final pathologic stage and histology; histology as an independent variable had many cells with small numbers

^d^Seven different uses of breast ultrasound (US) are shown in Table 1. On multivariate analysis, only the surgeon characteristic of “I perform breast US” was associated with fewer reexcisions

^e^Composite measure of any of the different methods of intraoperative margin assessment—gross pathologic, frozen section or touch prep cytology but excluding margin devices. Margin devices are commercial products available for intraoperative margin assessment such as but not limited to the MarginProbe™

^f^Specimen orientation not expected to impact reoperation rates. Purpose is to aid targeted re-excision in patients undergoing re-excision

### Outcome

The primary outcome was reoperation (either re-excision or mastectomy) within 90 days after initial BCS.

### Analyses

On preliminary review, a significant portion of missing data was associated with a small subset of seven participating physicians, who were excluded from the subsequent analyses. The remaining cases with missing data were included in the univariate (UV) but not in the MV analysis. Descriptive statistics were reported as proportions, medians (ranges), and means (standard deviations). Chi square, Fisher’s exact, and Wilcoxon rank-sum tests were used to compare demographic and clinical characteristics between patients who did and did not undergo reoperation. Factors that were significant on UV were included as candidate variables in the MV models. The preliminary MV model was constructed stepwise from the list of candidate variables, requiring *p* < 0.20 for initial inclusion and *p* < 0.10 for the variable to remain. The final mixed effects MV model was constructed by adding physician-level random intercepts. Expected rates of reoperation and physician contribution to the odds of reoperation were calculated using the final mixed effects MV model. All analyses were performed with the SAS 9.4 software suite (SAS Foundation, Cary, NC).

### Hierarchical Ranking

The relative strengths of association between explanatory variables and reoperation within 90 days were ranked by the F statistic, derived from the type III test of fixed effects (Table 2).

**Table 2:** Hierarchical ranking of patient, surgeon, tumor, and treatment factors for their effect on reoperations after breast conserving surgery for breast cancer

Effect	*F* value^c^	*p* value
Tumor focality (unifocal)	43.9	< 0.0001
Preoperative estimated tumor size (smaller)	22.0	< 0.0001
Neoadjuvant systemic therapy (receipt)^a^	8.5	0.0002
Composite measure pathologic tumor size and histology (smaller, invasive ductal)	7.6	< 0.0001
Pre-operative imaging (more than traditional 2D)^a,b^	6.1	0.0133
Surgeon practice type (solo/academic vs. group/hospital)	5.3	0.0013
Surgeon use of ultrasound (yes)^a,d^	5.3	0.0221
Intraoperative pathologist margin evaluation any type (gross/microscopic, but not device)^a^	4.9	0.0266
Patient age (> 80 yr)	4.5	0.0012
Physician case volume (top quartile)	4.3	0.0382
Cavity shaves (planned routine all sides)^a^	3.8	0.0219
Oncoplastic surgery (performed)^a^	3.7	0.0539
Primary insurance type (Medicare)	2.6	0.0231

^a^Actionable factor (under surgeon control)

^b^Pre-op imaging included one or more imaging modalities other than traditional 2D imaging (e.g., US, MRI, 3D mammography)

^c^The F-statistic is a measurement of the explanatory power of a given covariate to reoperations, after considering the effects of all other model covariates. To calculate the F-statistic for a specific covariate, the residual sum of squares for the full model is compared to the residual sum of squares for a model without the covariate in question. A larger F-statistic represents a larger contribution to the overall model’s explanatory power

^d^Surgeon performs US in their practice setting

### Data Validation

Seven surgeons (~ 10% of participants) were blinded to study co-investigators and randomly selected for a detailed audit. They voluntarily provided medical records for comparison to their previously self-reported data in Mastery^®^. Any discordances were reported to the surgeons in a password-protected format and were reconciled by supporting documentation. Cases that failed reconciliation were discarded.

The rate of discordance between surgeon entry and medical record review was 1.3% (34/2840). Following surgeon agreement, corrections were made in Mastery^®^ for these cases. No discordance occurred for documentation of reoperation.

## Results

**Fig. 2:**
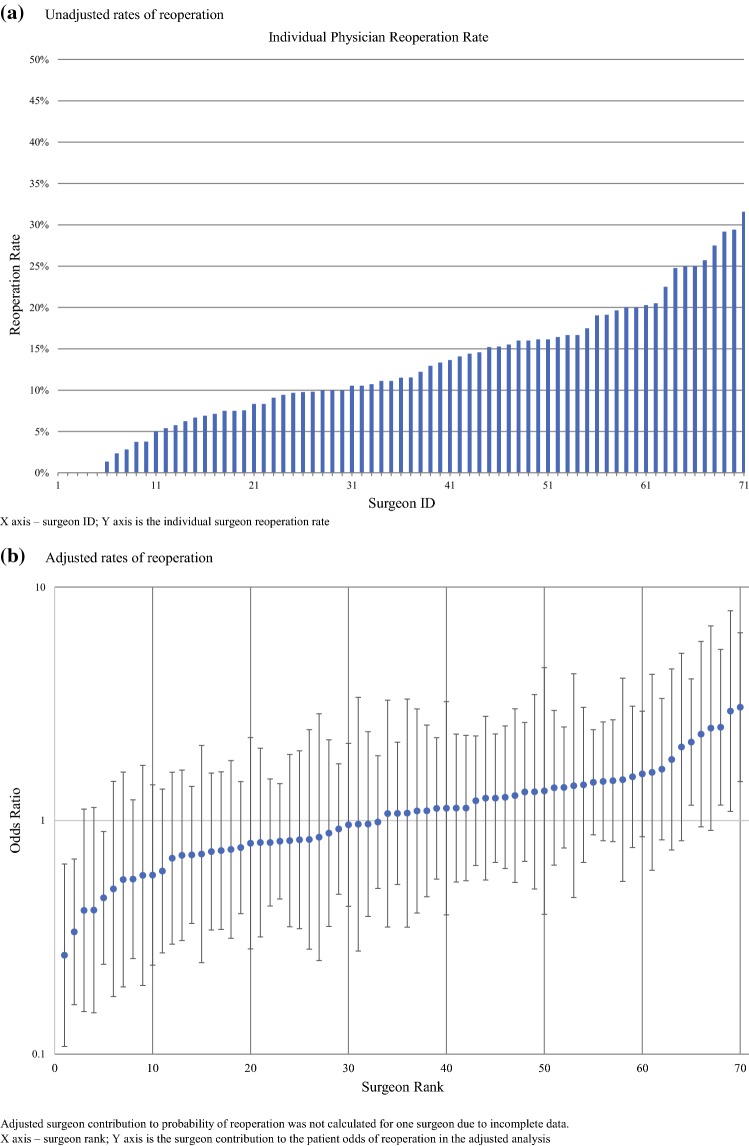
Intersurgeon variability of reoperation rates after initial breast conserving surgery for breast cancer

**Fig. 3:**
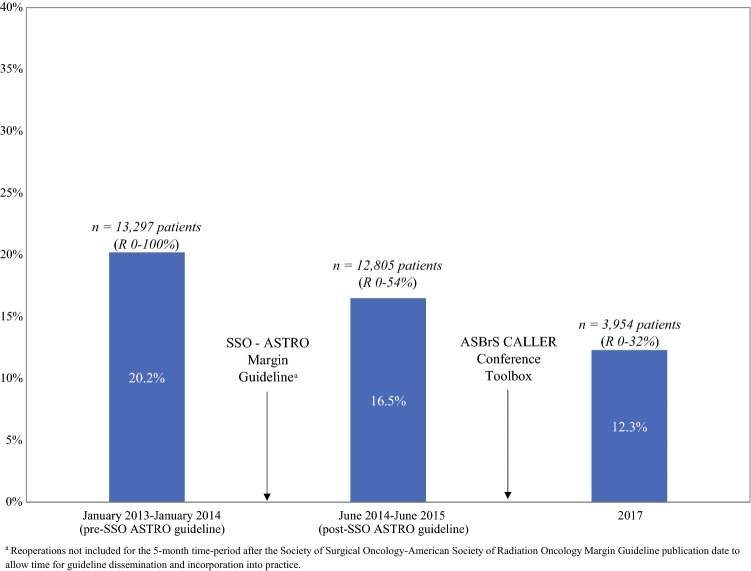
Overall reoperation rates after initial breast conserving surgery for cancer performed by American Society of Breast Surgeon (ASBrS) members entering cases into the ASBrS Mastery^®^ Patient Registry (2013-2017)

After excluding cases from surgeons with fewer than 10 cases/year, the overall reoperation rate for the remaining 71 surgeons was 12.3% (486/3954), mean 12.7% [SD 7.7%], median 11.5% [range 0–32%], unadjusted performance percentiles 3, 8, 12, 17, and 25% for the 10th, 25th, 50th, 75th, and 90th percentiles [10th–90th difference = 8.3X; IQR 0.17–0.08]. The per annum case volume of surgeons in the highest quartile ranged from 74 to 180 (mean 115, median 107). Their unadjusted reoperation rates were 10.6% compared with 13.9% for lower-volume surgeons. Thirty-one surgeons (44%) had rates of 10% or less. The reoperation rate in five surgeons with < 10 cases in 2017 was 3.2% (1/31). The association of all covariates with reoperation, intersurgeon variability and ASBrS member rates over time are shown in Table 1 and Figs. 2 and 3. Of seven different uses of breast ultrasound, only the breast surgeon characteristic of “I preform breast US” was associated with fewer reoperations.

In 130 cases of reexcision after BCS for DCIS, the reasons for reexcision (by margin status) were ink + in 59 (45.4%), < 1 mm in 55 (42.4%), 1–2 mm in 14 (10.8%), and “other” causes in 2 (1.6%) cases. For 349 cases with invasive cancer (± synchronous DCIS), the reasons for reexcision (by margin status) were ink + in 248 (71.1%), < 1 mm in 63 (18.1%), 1–2 mm in 22 (6.3%), and other causes in 16 (4.6%).

The hierarchical ranking of those factors associated with fewer reoperations were, in descending order, tumor focality (unifocal), estimated tumor size (smaller), neoadjuvant systemic therapy (receipt), a composite measure of pathologic tumor size and histology (smaller, invasive ductal), use of a preoperative diagnostic imaging modality beyond conventional 2D mammography, surgeon practice type (solo/academic versus group/hospital), surgeon use of ultrasound (US) [in their practice setting], intraoperative pathologic margin assessment [any type other than margin devices], patient age (> 80), surgeon case volume (highest quartile), cavity side-wall shaves (routine planned), and insurance type (Medicare; Table 2).

## Discussion

Variation of reoperation rates after BCS for cancer represents an opportunity for process improvement at both the surgeon and facility level. Recognizing high rates and variation as early as 2012, a “plan-do-study-act” performance improvement project was initiated by the ASBrS the following year.^29^

This initiative included ranking and specifying reoperation rates [along with other domains of care], incorporating a reoperation metric into the ASBrS patient registry for auditing, and providing a web-based platform for benchmarking.^30^ During benchmarking, surgeons compared their personal rates [in real-time] to the de-identified rates of all other ASBrS surgeons entering data. Two planned data reviews indicated persistently high rates and inter-surgeon variability.^4^,^6^ Further action plans to reduce rates were therefore undertaken. First, a national multidisciplinary consensus conference (CALLER) was convened to endorse ten specific processes of care, or “tools,” found by others to be associated with fewer reoperations.^18^ This toolkit was then communicated to other surgeons by publication and by presentation at the national ASBrS meeting. Second, after the CALLER consensus conference, new data fields were incorporated into the ASBrS patient registry to capture receipt of which endorsed tools were used for each patient undergoing an initial BCS. Another planned prospective analysis of sequential patients undergoing BCS for 1 year followed, and we report the results of this analysis here. We sought to determine which of the tools endorsed by the ASBrS in 2015 were utilized by its members in 2017 and which were associated with fewer reoperations after BCS.

In 2013 and 2015, the rates of reoperation were 21.6% and 16.5% respectively for ASBrS members participating in Mastery^®^.^4^,^6^ In the current study year (2017), the overall rate of reoperation by 71 surgeons was 12.3% (Fig. 3). To our knowledge, this is the lowest rate yet identified in a national registry. Furthermore, more than one in three surgeons achieved the ASBrS recommended target goal of 10%.^18^ Of note, the highest rate in the current study of 32% is a very low “peak” rate compared with other publications.^1^^–^8,10^–^16 However, significant intersurgeon variability was identified, and there was greater than a eightfold difference between surgeons at the 10th and 90th performance percentiles. Thus, future efforts to reduce variability are still indicated. We are unaware of any professional organization recommending use of a specific reoperation target goal for accountability purposes, such as for pay for performance incentives or patient steerage.

### Opportunities

The *actionable* processes of care associated with fewer reoperations in the current study were, in descending order of influence, receipt of neoadjuvant chemotherapy (NAC), use of a preoperative diagnostic imaging modality beyond conventional 2D mammography, surgeon use of ultrasound (US), intraoperative pathologic [margin] assessment [of any type other than margin devices], and routine planned cavity side-wall shaves (Tables 1 and 2). These findings are consistent with selected prior randomized trials, meta-analyses, and observational studies.^18^,^31^^–^40 Thus, all are recommended for consideration of adoption by breast surgeons when appropriate given a patient’s presentation and tumor subtype. Of note, selective cavity shavings (based on intraoperative findings) were not associated with fewer reoperations. Selective shaving is a different process compared to a priori planned all side-wall shaves, as demonstrated to be successful in two randomized trials.^31^,^32^

For some tools, such as cavity shaves and NAC, there are few barriers to implementation. For patients in whom NAC is appropriate based on the tumor type, size, and nodal status, there are additional benefits beyond reducing reoperations. NAC de-escalates the overall chance of mastectomy and axillary dissections, especially for patients with subtypes known to have high response rates.^41^^–^44 The use of intraoperative imaging with US has not always been available to the vast majority of surgeons performing breast surgery. The ASBrS has addressed this concern by providing education and certification programs.^45^ Our findings of fewer reoperations with surgeon use of US reinforce the importance of the ASBrS US training and accreditation programs.

For each of the processes described, there are opportunities for improvement based on the frequencies of their use by ASBrS members (Table 1). For example, planned cavity shaves—a procedure that requires no equipment and adds minimal additional time to the length of the procedure—were utilized in only 1334 (34%) of 3942 cases. For those surgeons with a higher than average rate of reoperation, incorporating this tool into their standard operative approach could be advised. Adoption of every process of care shown here to be associated with fewer reoperations is not recommended. The selection of which to adopt will depend on patient factors, surgeon setting, resource availability, and facility-specific barriers. If rates are at or below target goals with current practices, then adopting new processes may not be value-added.

Another opportunity for improvement identified in the current study is increased compliance with the SSO-ASTRO guideline.^20^ This guideline was based on a meta-analysis that demonstrated that reexcisions wider than no-ink on tumor did not lessen in-breast cancer recurrence in patients with invasive disease.^19^ After reviewing reasons for reexcision in patients with invasive cancer in the current study, we found that more than 20% of reexcisions were performed in patients with an ink-negative margin. In contrast, surgical care was guideline-compliant with the 2016 margin guideline for DCIS.^21^ Reexcision for margins > 2 mm rarely occurred.

Some factors associated with reoperations are immutable; i.e., they are not modifiable upon patient presentation and therefore not actionable to improve rates. These include tumor histology, tumor focality, and surgeon practice type. There were fewer reoperations in solo- and academic-practice surgeon settings compared with other settings, and rates were higher with lobular histology and multifocality. Tumor size also is fixed at patient presentation; however, in select eligible patients, NAC can be considered and is associated with fewer reoperations.^35^,^36^ Lastly, commercial insurance, compared with no or “other” insurance, was another fixed factor associated with higher rates. Wilke et al. also reported an association between insurance type and reoperation rates in the NCDB; no coverage was associated with the lowest rates.^3^ The association between reimbursement incentives, provider practice, surgical volume, and surgical outcomes is complex and requires greater depth of information than what is provided in this patient registry.^46^

### Factors not Associated with Reoperations

Notable factors not associated with fewer reoperations included patient race, preoperative breast magnetic resonance imaging (MRI), and the method of tumor localization. A recent meta-analysis also demonstrated no improvement in reoperations with MRI.^47^ Other processes of care were investigated, because recent studies had demonstrated their effectiveness in reducing reoperations. For example, two randomized trials demonstrated fewer reoperations with the MarginProbe^®^ device.^48^,^49^ In our analysis, reoperation rates with and without the device [9.9% and 12.5%; *p* = 0.280] did not achieve statistical significance. Onco-plastic surgery also has been associated with fewer positive margins and reoperations in other studies.^33^,^50^^–^52 In the current study, the unadjusted rates with and without receipt of it were 10.4% and 13.2% (*p* = 0.017). After adjustment, the odds ratio was 0.76 [95% CI 0.57–1.00; *p* = 0.054]. If positive margins occur after oncoplastic surgery, identification of the location for reexcision may be challenging.

### Frequencies of Use of Diagnostic Modalities and Processes of Care

Determining the frequency of use of various processes of care within the CALLER toolbox to decrease reoperations was not the primary focus of the current study; however, they are shown in Table 1 and reflect the distribution of their use in a contemporary cohort of surgeons that collectively had a very low rate of reoperation. The profile of the processes of care that they employ may differ from non-ASBrS member surgeons.

Study strengths included prospective data entry, a very low error rate of surgeon-entered data, a sample size larger than that in five recent publications comparing reoperation rates before and after the SSO-ASTRO margin guideline, and collection of covariates beyond the typical patient and tumor factors used for risk adjustment.^9^^–^14 Additionally, those processes of care that are important predictors for reoperations, such as surgeon use of US, cavity shaves, and reasons for reoperation (including margin status), were able to be captured. This granularity of information is not available in other commonly used national data sets.

### Limitations

All patient registry studies can have unmeasured confounders that introduce bias. We limited this risk by including covariates for processes of care. Risk adjustment in many past investigations was restricted to patient, tumor, and treatment characteristics. Also, we were unable to explain the reasons why surgeons who perform breast US have fewer reoperations.

The generalizability of the current study findings to other surgeons is unknown. The low rate of reoperations demonstrated here may not reflect other surgeon groups. Voluntary participation may have preferentially captured a dedicated group of surgeons with a focus on quality improvement. On the other hand, all the processes of care that we found to be associated with fewer reoperations in this seemingly “exceptional” group of surgeons are potentially available to all breast surgeons.

Our goal is to reduce intersurgeon variability by simple adoption of those tools found to be associated with lower rates. As a cautionary note, we advise that surgeons continue guideline-compliant care to re-excise in patients with positive margins and to continue to offer breast conservation to all eligible patients. Failing to re-excise a positive margin by a surgeon not wanting to report a reoperation would be expected to result in higher rates of cancer recurrence.

## Conclusions

To provide a snapshot of the efficacy of those processes of care endorsed by a 2015 consensus conference to lower reoperation rates, 71 member-surgeons of the ASBrS entered data on nearly 4000 patients undergoing BCS for cancer.

Surgeon use of US, routine planned cavity side-wall shaves, and NAC were associated with fewer reoperations. Other opportunities for improvement were identified as well, specifically by increasing compliance with the SSO-ASTRO margin guideline for invasive cancer.

A low overall rate of reoperation (12.3%) was found, but variability persisted. As surgeons and facilities increase participation in benchmarking programs and regional and state collaborative improvement programs expand, further reductions in variability are likely to occur.
